# Detection of secondary upper gastrointestinal tract cancer during follow‐up esophagogastroduodenoscopy after gastrectomy for gastric cancer

**DOI:** 10.1002/ags3.12546

**Published:** 2022-01-25

**Authors:** Kosuke Nakane, Keiichi Fujiya, Masanori Terashima, Takanori Kawabata, Yosuke Matsumoto, Satoshi Kamiya, Makoto Hikage, Yutaka Tanizawa, Hiroyuki Ono, Etsuro Bando

**Affiliations:** ^1^ Division of Gastric Surgery Shizuoka Cancer Center Nagaizumi Japan; ^2^ Clinical Research Center Shizuoka Cancer Center Nagaizumi Japan; ^3^ Division of Endoscopy Shizuoka Cancer Center Nagaizumi Japan

**Keywords:** Fine and Gray model, follow‐up EGD, gastrectomy, remnant gastric cancer, secondary cancer

## Abstract

**Aim:**

Esophagogastroduodenoscopy (EGD) may contribute to early detection of secondary cancer in the upper gastrointestinal tract although the clinical relevance of follow‐up after gastrectomy remains unclear. This study aimed to elucidate the effectiveness of follow‐up EGD by investigating the incidence of secondary cancer in any part of the upper gastrointestinal tract.

**Methods:**

Data from 1438 patients who underwent curative partial gastrectomy for primary gastric cancer between 2008 and 2014 and follow‐up EGD at least once during a 5‐year follow‐up period were retrospectively reviewed. Incidence rates of remnant gastric cancer, laryngeal cancer, and esophageal cancer detected after follow‐up EGD were determined, and risk factors for secondary cancers were examined. The characteristics of clinicopathological diagnoses of secondary cancers were reviewed and compared according to the frequency of follow‐up EGD.

**Results:**

The average annual frequency of EGD was 0.7, while the 5‐year cumulative incidence rates of remnant gastric cancer and secondary laryngeal and esophageal cancers were 2.9% and 1.3%, respectively. Risk factors for remnant gastric cancer included heavy smoking, proximal gastrectomy, and tumor size ≥ 30 mm. All secondary cancers were resectable upon diagnosis, with endoscopically resectable cancer accounting for 81.0% of cases. Our results found a significantly higher proportion of endoscopically resectable cancers during regular follow‐up than during infrequent follow‐up.

**Conclusions:**

Follow‐up EGD can be a useful modality for detecting secondary upper gastrointestinal tract cancer, likely leading to curative treatment for secondary cancer. Focusing on patients presenting with risk factors may increase the value of follow‐up EGD after gastrectomy.

## Graphical abstract

Cumulative incidence rates of secondary upper gastrointestinal cancer, remnant gastric cancer and laryngeal and esophageal cancer at 5 year after surgery are 4.2%, 2.9% and 1.3%, respectively.

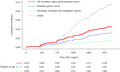

## 1 INTRODUCTION

Curative resection is the standard treatment for gastric cancer, and surgery combined with adjuvant chemotherapy improves survival rates.^1^ However, the significance of and optimum protocol for follow‐up after surgery remain unclear. Follow‐up after gastrectomy is generally conducted to diagnose post‐gastrectomy syndromes and postoperative complications, and promptly detect secondary cancer and recurrence.^2^ Some studies have shown that early detection of secondary cancer or recurrence during follow‐up after gastrectomy improved overall survival; however, other studies have shown that early detection of recurrence after intensive follow‐up examinations did not improve overall survival.^3^, ^4^, ^5^

Five‐year cumulative incidence rates of remnant gastric cancer after gastrectomy have been estimated in the range of 1.4%‐6.8%,^6^, ^7^, ^8^ with patients having early remnant gastric cancer showing relatively good survival outcomes.^9^, ^10^, ^11^ Esophagogastroduodenoscopy (EGD) is useful for the detection of early remnant gastric cancer,^10^ and the 2018 Japanese Gastric Cancer Treatment Guidelines recommend biennial follow‐up EGD.^2^ Follow‐up EGD may increase the probability of early disease detection and curative treatment for remnant gastric cancer,^5^, ^6^, ^12^ helping improve outcomes.

Although several studies on follow‐up EGD and remnant gastric cancer are available, only a few studies have investigated secondary cancers in the upper gastrointestinal tract. Patients with gastric cancer may have a high risk of secondary head and neck cancers and esophageal cancers.^13^ We examined the incidence rates of secondary cancer in the entire upper gastrointestinal tract to evaluate the effectiveness of follow‐up EGD in detecting secondary cancer. Early diagnosis of secondary cancer may enable the administration of less invasive curative treatment and improve survival. Moreover, elucidating risk factors for secondary cancer may help identify patients requiring intensive follow‐up examinations.

This study aimed to evaluate the effectiveness of regular follow‐up EGD after gastrectomy by analyzing 5‐year cumulative incidence rates of secondary cancer in any part of the upper gastrointestinal tract and associated treatment outcomes. Moreover, risk factors for secondary cancer and characteristics of patients most likely to benefit from follow‐up EGD were identified.

## 2 METHODS

### 2.1 Patients

**FIGURE 1:**
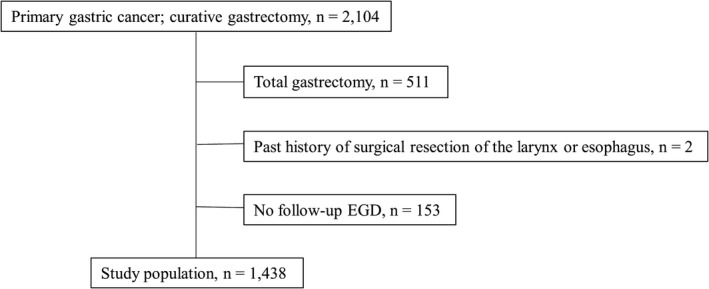
Flow chart of patient enrollment. EGD, esophagogastroduodenoscopy

A total of 2104 patients underwent curative gastric resection (R0 surgery) for primary gastric cancer between January 2008 and December 2014 at the Shizuoka Cancer Center. A total of 1438 patients were included after 511, two, and 153 patients who underwent total gastrectomy, had a history of surgical resection of the larynx or esophagus, and had not undergone EGD within 5 years after gastrectomy, respectively, were excluded (Figure 1).

### 2.2 Follow‐up period and EGD examination

According to the 2018 Japanese Gastric Cancer Treatment Guidelines,^2^ EGD examination was performed 1, 3, and 5 years after surgery, although the timing of EGD and medical facilities where patients received EGD were determined for each patient by the attending surgeons. Data on EGD examinations performed at the clinic were extracted from medical records, patient referral documents, and EGD reports. Atrophic gastritis was evaluated using the Takemoto‐Kimura classification,^14^ with moderate or severe atrophic gastritis defined as C‐3, O‐1, O‐2, and O‐3.

### 2.3 Definition of secondary upper gastrointestinal cancer

The present study included patients with lower laryngeal cancer as part of the upper gastrointestinal tract cancers. Lower laryngeal cancer and esophageal cancer detected during the follow‐up period, including intraepithelial neoplasia, were defined as secondary laryngeal and esophageal cancers. Remnant gastric cancer and secondary laryngeal and esophageal cancers were defined as secondary upper gastrointestinal cancers. Staging of each cancer was based on the Union for International Cancer Control TNM classification of malignant tumors, 8th edition.^15^

### 2.4 Cumulative incidence rates, risk factors, and treatment

Cumulative incidence rates of and risk factors for remnant gastric cancer and secondary laryngeal and esophageal cancers were examined.

Secondary cancer was classified as endoscopically resectable cancer, according to the 2018 Japanese Gastric Cancer Treatment Guidelines,^2^ and surgically resectable cancer and unresectable cancer, based on clinicopathological diagnosis. Patients who underwent surgery for secondary cancer were followed up for an additional 5 years after secondary cancer diagnosis.

### 2.5 Statistical analyses

The Fisher exact test and Mann‐Whitney *U* test were used to compare categorical and continuous variables between groups, respectively. We regarded death as a competing risk in the analysis of secondary upper gastrointestinal cancer cumulative incidence rates. Risk factors for secondary cancer were analyzed using the Fine and Gray model,^16^ in which the strength of the association between variables and secondary cancer risk was assessed using the subdistribution hazard ratio (SHR). Risk factors were examined in the multivariate analysis using variables with *P*‐values of <.1 in the univariate analysis. All statistical analyses were performed using R Statistics version 4.0.1 (http://www.r‐project.org/), with two‐sided *P*‐values of <.05, indicating statistical significance.

## 3 RESULTS

### 3.1 Follow‐up EGD examination

The median follow‐up duration for EGD was 1700 days (25^th^‐75^th^ percentile, 1452‐1829 days), while the average frequency of EGD was 0.7 times per year. The implementation rates of EGD per year after surgery were 74.6%, 60.9%, 71.0%, 66.6%, and 70.6% in the first, second, third, fourth, and fifth years, respectively. Approximately 30% of the patients received EGD annually, while approximately 70% received EGD at intervals of no more than 2 years. A total of 4684 EGD examinations were performed in all patients: 3513 (75.0%) at our hospital and 1171 (25.0%) at the clinic.

### 3.2 Clinicopathological characteristics

The clinicopathological characteristics of the patients at initial surgery are summarized in Table 1. Forty‐three patients had a history of upper gastrointestinal cancer, and all previous upper gastrointestinal cancers were curatively treated using endoscopy or chemoradiotherapy.

**TABLE 1:** Clinicopathological characteristics of patients

	Total	Secondary upper gastrointestinal cancer		*P*
		Yes	No	
	n = 1438	n = 64	n = 1374	
Sex				
Male	963 (67.0%)	56 (87.5%)	907 (66.0%)	<.001
Female	475 (33.0%)	8 (12.5%)	467 (34.0%)	
Age (years)				
≥70	561 (39.0%)	26 (40.6%)	535 (38.9%)	.794
<70	877 (61.0%)	38 (59.4%)	839 (61.1%)	
Brinkman index				
≥600	531 (36.9%)	39 (60.9%)	492 (35.8%)	<.001
<600	907 (63.1%)	25 (39.1%)	882 (64.2%)	
Alcohol				
Habitual drinker	713 (49.6%)	37 (57.8%)	676 (49.2%)	.201
Nondrinker or social drinker	725 (50.4%)	27 (42.2%)	698 (50.8%)	
History of gastric cancer before gastrectomy				
Yes	25 (1.7%)	4 (6.3%)	21 (1.5%)	.022
No	1413 (98.3%)	60 (93.8%)	1353 (98.5%)	
History of esophageal cancer				
Yes	19 (1.3%)	6 (9.4%)	13 (0.9%)	<.001
No	1419 (98.7%)	58 (90.6%)	1361 (99.1%)	
Preoperative chemotherapy				
Yes	18 (1.3%)	0 (0.0%)	18 (1.3%)	1.000
No	1420 (98.7%)	64 (100.0%)	1356 (98.7%)	
Atrophic gastritis before initial surgery				
Moderate or severe (C‐3, O‐1, O‐2, O‐3)	1174 (81.6%)	61 (95.3%)	1113 (81.0%)	.002
Mild or no atrophy (C‐0, C‐1, C‐2)	264 (18.4%)	3 (4.7%)	261 (19.0%)	
Synchronous multiple gastric cancer				
Yes	185 (12.9%)	12 (18.8%)	173 (12.6%)	.178
No	1253 (87.1%)	52 (81.3%)	1201 (87.4%)	
Surgical procedure				
Distal gastrectomy	1048 (72.9%)	44 (68.8%)	1004 (73.1%)	.028
Proximal gastrectomy	99 (6.9%)	10 (15.6%)	89 (6.5%)	
Pylorus preserving gastrectomy	291 (20.2%)	10 (15.6%)	281 (20.5%)	
Pathohistology of main initial cancer				
Differentiated type	753 (52.4%)	41 (64.1%)	712 (51.8%)	.155
Undifferentiated type	660 (45.9%)	23 (35.9%)	637 (46.4%)	
Special type	25 (1.7%)	0 (0.0%)	25 (1.8%)	
Tumor size				
≥30 mm	947 (65.9%)	44 (68.8%)	903 (65.7%)	.687
<30 mm	491 (34.1%)	20 (31.3%)	471 (34.3%)	
Depth of tumor invasion				
T0/T1a	424 (29.5%)	14 (21.9%)	410 (29.8%)	.435
T1b	580 (40.3%)	26 (40.6%)	554 (40.3%)	
T2	162 (11.3%)	8 (12.5%)	154 (11.2%)	
T3	166 (11.5%)	8 (12.5%)	158 (11.5%)	
T4a	100 (7.0%)	8 (12.5%)	92 (6.7%)	
T4b	6 (0.4%)	0 (0.0%)	6 (0.4%)	
Lymphatic metastasis				
Positive	430 (29.9%)	17 (26.6%)	413 (30.1%)	.675
Negative	1008 (70.1%)	47 (73.4%)	961 (69.9%)	
Pathological stage				
0‐I	1042 (72.5%)	44 (68.8%)	998 (72.6%)	.357
II	255 (17.7%)	10 (15.6%)	245 (17.8%)	
III	139 (9.7%)	10 (15.6%)	129 (9.4%)	
IV	2 (0.1%)	0 (0.0%)	2 (0.2%)	
Adjuvant chemotherapy				
Yes	267 (18.6%)	9 (14.1%)	258 (18.8%)	.413
No	1171 (81.4%)	55 (85.9%)	1116 (81.2%)	

### 3.3 Cumulative incidence rates and characteristics of secondary upper gastrointestinal cancer

**FIGURE 2:**
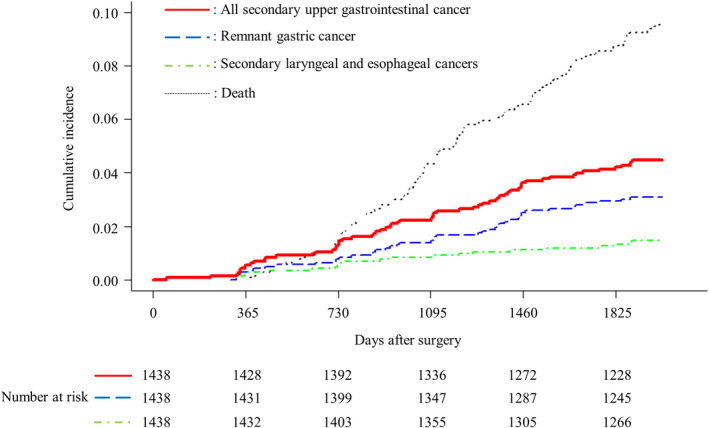
Cumulative incidence rates of secondary upper gastrointestinal cancer

Remnant gastric cancer, lower laryngeal cancer, and esophageal cancer were found in 44, eight, and 15 patients, respectively. Remnant gastric cancer and lower laryngeal cancer were found in one patient, and laryngeal cancer and esophageal cancer were diagnosed in two patients during the same EGD examination. The cumulative incidence rates of remnant gastric cancer at the first, third, and fifth year after surgery were 0.3%, 1.4%, and 2.9%, respectively, and those of secondary laryngeal and esophageal cancers were 0.3%, 0.8%, and 1.3%, respectively (Figure 2).

A single patient presented with sore throat at the time of lower laryngeal cancer and esophageal cancer diagnosis; asymptomatic cancers were detected during follow‐up EGD in the remaining 63 patients. No other modalities, including tumor markers, ultrasonography, or computed tomography, triggered the detection of secondary cancers.

The clinicopathological characteristics of patients with or without secondary upper gastrointestinal cancer are summarized in Table 1. Proximal gastrectomy was significantly more frequent in patients with secondary upper gastrointestinal cancer than in those who underwent other surgical procedures (*P* = .010).

### 3.4 Risk factors

The risk factors for remnant gastric cancer are shown in Table 2. Our analysis identified a Brinkman index ≥ 600 (SHR 2.243, 95% confidence interval [CI] 1.121‐4.487, *P* = .022), proximal gastrectomy (SHR 3.925, 95% CI 1.887‐8.165, *P* < .001), and tumor size ≥ 30 mm (SHR 3.013, 95% CI 1.328‐6.836, *P* = .008) as risk factors for remnant gastric cancer. The 5‐year cumulative incidence rates of remnant gastric cancer in patients with a Brinkman index ≥ 600, who underwent proximal gastrectomy, and with a tumor size ≥ 30 mm were 5.0%, 9.2%, and 3.6%, respectively (Figure S1). Risk factors for secondary laryngeal and esophageal cancers included a history of gastric cancer (SHR 4.937, 95% CI 1.630‐14.950, *P* = .005) and esophageal cancer (SHR 17.736, 95% CI 6.533‐48.153, *P* < .001) before gastrectomy (Table S1).

**TABLE 2:** Risk factors for remnant gastric cancer

	Number of patients	Univariate analysis	*P*	Multivariate analysis	*P*
		Subdistribution hazard ratio (95% CI)		Subdistribution hazard ratio (95% CI)	
Sex					
Female	475	1	.018	1	.437
Male	963	2.650 (1.183‐5.938)		1.454 (0.565‐3.742)	
Age (years)					
<70	877	1	.217		
≥70	561	1.451 (0.804‐2.621)			
Brinkman index					
<600	907	1	<.001	1	.022
≥600	531	2.778 (1.515‐5.093)		2.243 (1.121‐4.487)	
Alcohol					
Nondrinker or social drinker	725	1	.586		
Habitual drinker	713	0.848 (0.469‐1.535)			
History of gastric cancer before gastrectomy					
No	1413	1	.782		
Yes	25	1.327 (0.179‐9.856)			
Atrophic gastritis before initial surgery					
Mild or no atrophy (C‐0, C‐1, C‐2)	264	1	.031	1	.054
Moderate or severe (C‐3, O‐1, O‐2, O‐3)	1174	4.754 (1.152‐19.615)		4.034 (0.977‐16.660)	
Synchronous multiple gastric cancer					
No	1253	1	.291		
Yes	185	1.508 (0.703‐3.233)			
Surgical procedure					
Distal gastrectomy/Pylorus preserving gastrectomy	1339	1	<.001	1	<.001
Proximal gastrectomy	99	3.581 (1.727‐7.427)		3.925 (1.887‐8.165)	
Pathohistology of main initial cancer					
Differentiated type	753	1	.036	1	.335
Undifferentiated/Special type	685	0.508 (0.270‐0.957)		0.727 (0.380‐1.391)	
Tumor size					
<30 mm	491	1	.029	1	.008
≥30 mm	947	2.353 (1.093‐5.067)		3.013 (1.328‐6.836)	
Depth of tumor invasion					
T0/T1	1004	1	.122		
T2/T3/T4	434	1.607 (0.881‐2.929)			
Lymphatic metastasis					
Negative	1008	1	.953		
Positive	430	0.981 (0.514‐1.872)			
Pathological stage					
0‐I	1042	1	.525		
II‐IV	396	1.228 (0.652‐2.313)			
Adjuvant chemotherapy					
No	1171	1	.384		
Yes	267	0.683 (0.290‐1.611)			

Abbreviation: CI, confidence interval.

### 3.5 Pathological staging

**FIGURE 3:**
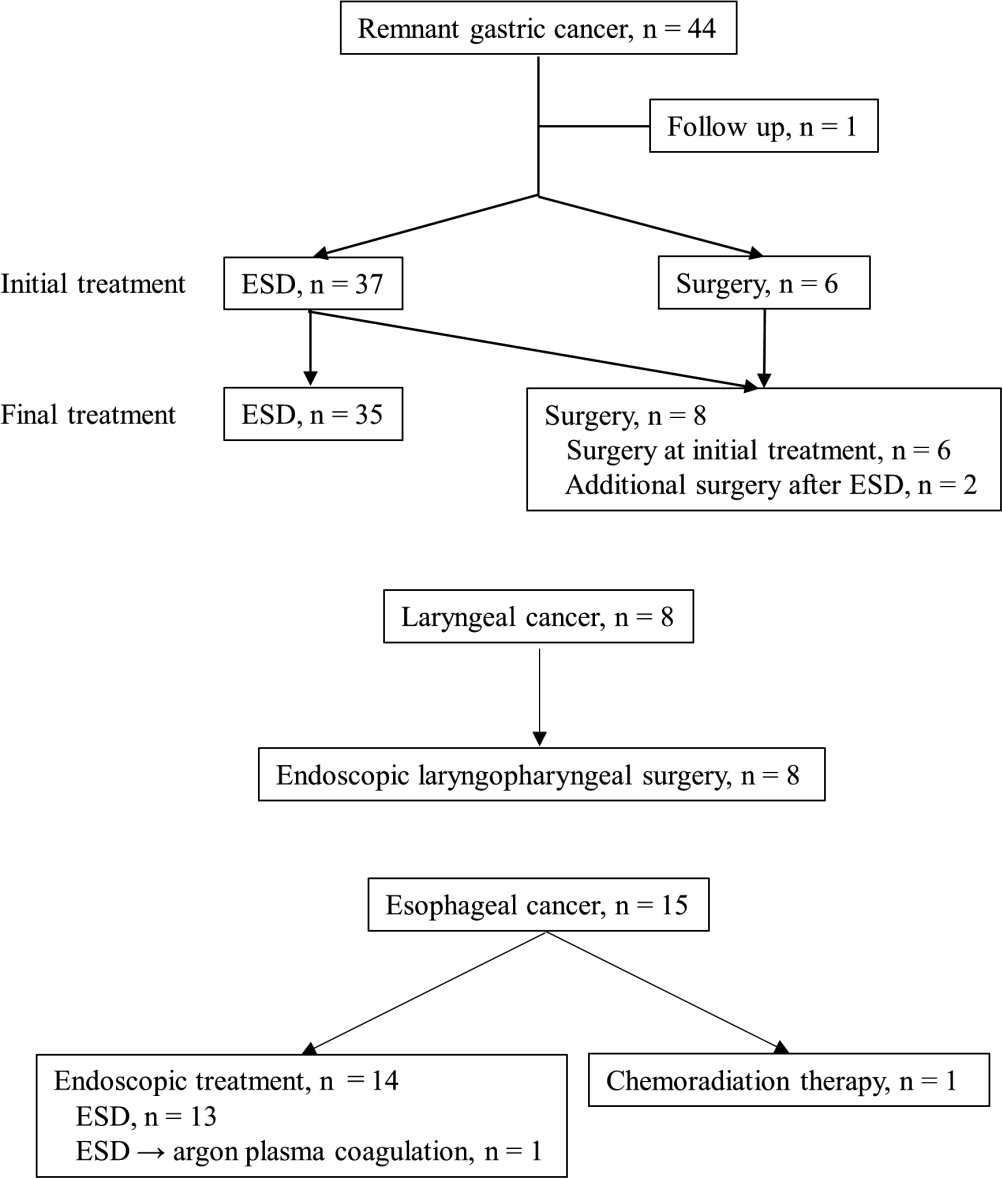
Flow chart of the secondary upper gastrointestinal cancer treatment. ESD, endoscopic submucosal dissection

The treatment for secondary upper gastrointestinal cancer is shown in Figure 3. Among 44 patients with remnant gastric cancer, 40 and four had clinical T1 and T2‐4 tumors, respectively. All remnant gastric cancers were resectable upon diagnosis. Final treatments included endoscopic submucosal dissection (ESD) and surgery in 35 and eight patients, respectively. One patient was followed up without any treatment owing to old age, comorbidities, and low performance status. Resected cancers were at stages I, II, and III in 39, two, and two patients, respectively; all 43 patients underwent curative treatment. Pathologically, endoscopic curative resectable cancers were observed in 33 (76.7%) patients.

Among 15 patients with esophageal cancer, 13 had clinical Tis or T1a tumors, the remaining two had clinical T1b tumors, and one developed lymphatic metastasis. ESD, argon plasma coagulation, and curative chemoradiotherapy were performed in 13 patients with clinical Tis–T1a, a single patient with clinical T1bN0, and another patient with clinical T1bN1. Endoscopic curative resection was performed in all 13 patients who underwent ESD. Laryngeal cancers in eight patients were diagnosed with clinical Tis and curatively resected via endoscopic laryngopharyngeal surgery.

**FIGURE 4:**
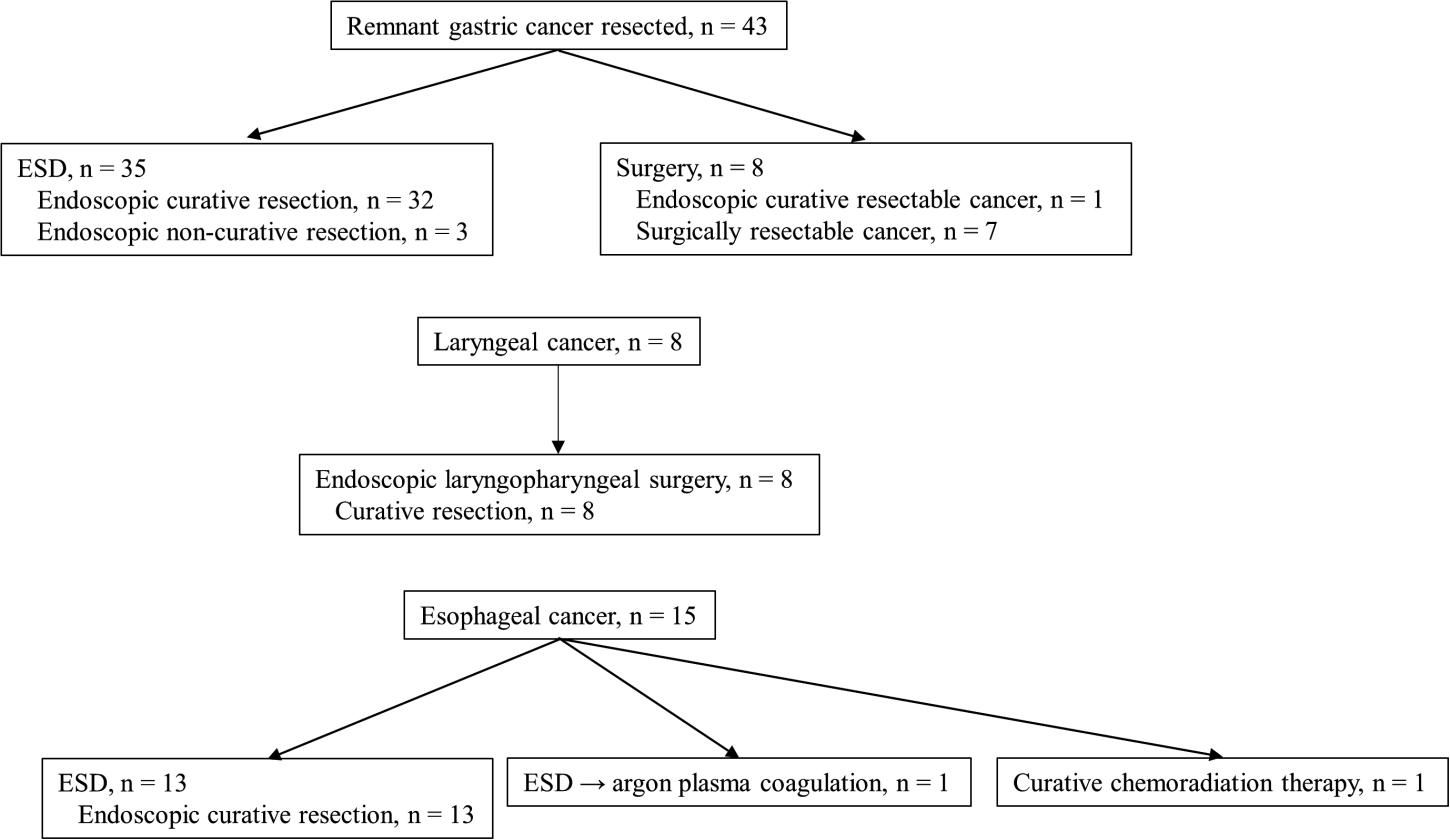
Pathological staging of secondary upper gastrointestinal cancer. ESD, endoscopic submucosal dissection

Pathological staging findings of each cancer type are summarized in Figure 4. All cancers were resectable, with endoscopically resectable cancer accounting for 51 (81.0%) of the 63 patients who underwent curative treatment.

### 3.6 Survival outcomes

All 1438 patients had 1‐, 3‐, and 5‐year overall survival rates of 99.9%, 95.5%, and 91.0%, respectively. Among 139 patients who died during the follow‐up period, 59 (42.4%) and two (1.4%) died due to initial gastric cancer and secondary upper gastrointestinal cancer, respectively. Of the remaining 78 patients, 25 died of other cancers, 43 died of other diseases, two died in an accident, and the cause of death was unknown in eight. Recurrence of remnant gastric cancer was found in four patients with pathological stage II or stage III remnant gastric cancer. No recurrence of secondary upper gastrointestinal cancer was found in the other patients.

### 3.7 Treatment and cancer progression according to frequency of follow‐up EGD

Among the included patients, 1016 (70.7%) who underwent EGD ≥ 3 times in 5 years (annual frequency ≥ 0.6) were categorized into the regular follow‐up group, while the remaining 422 (29.3%) patients were categorized into the infrequent follow‐up group. The regular and infrequent follow‐up groups had an average annual follow‐up EGD frequency of 0.8 and 0.3, respectively. The infrequent follow‐up group included more individuals aged ≥70 years (48.3%) than the regular follow‐up group (35.1%). Moreover, the infrequent follow‐up group showed higher proportions of differentiated adenocarcinoma, pathological T2‐T4 tumors, and lymphatic metastasis of initial gastric cancer (57.1%, 34.4%, and 34.6%, respectively) than the regular follow‐up group (50.4%, 28.4%, and 28.0%, respectively). No significant differences in other patient characteristics were observed (Table S2).

Secondary upper gastrointestinal cancer was found in 48 and 16 patients in the regular and infrequent follow‐up groups, respectively. Among 64 patients with secondary upper gastrointestinal cancer, the regular follow‐up group had a significantly higher proportion of habitual drinkers (66.7%) than the infrequent follow‐up group (31.3%), although no significant differences in other patient characteristics were noted (Table S3). Cumulative incidence rates of secondary upper gastrointestinal cancer at the first, third, and fifth year after surgery were 0.7%, 2.5%, and 4.6% in the regular follow‐up group and 0.0%, 1.7%, and 3.3% in the infrequent follow‐up group, respectively (Figure S2). No significant difference in the cumulative incidence rates was observed between the groups (SHR, 1.261; 95% CI, 0.713‐2.216; *P* = .421).

Among 63 patients who received treatment, 42 (87.5%) and nine (60.0%) in the regular and infrequent follow‐up groups had endoscopically resectable cancer, respectively (Table S4; *P* = .028). Recurrence of remnant gastric cancer was found in two patients in each group (*P* = .238).

## 4 DISCUSSION

Among 1438 patients who underwent gastrectomy, 2.9% were found to have remnant gastric cancer, while 1.3% developed laryngeal and esophageal cancer. All secondary cancers were resectable, and most of them underwent curative endoscopic resection.

One study on EGD screening for the general Japanese population showed a 0.8% overall 5‐year cumulative incidence rate of gastric cancer,^17^ and the present study indicated relatively higher incidence rates of remnant gastric cancer after gastrectomy. Our analyses identified a Brinkman index ≥ 600, proximal gastrectomy, and tumor size ≥ 30 mm as risk factors for remnant gastric cancer. Each risk factor was associated with 5‐year cumulative remnant gastric cancer incidence rates in the range of 3.6%‐9.2%, suggesting a need for modifying EGD follow‐up intervals, depending on the combination of observed risk factors.

Approximately 70% of the patients underwent follow‐up EGD ≥ 3 times in 5 years (annual frequency ≥ 0.6), as recommended by the 2018 Japanese Gastric Cancer Treatment Guidelines. In Japan, gastric cancer screening is recommended once every 2 years, while the frequency of follow‐up EGD after gastrectomy for gastric cancer is almost the same as that for gastric cancer screening.

Reports have shown 5‐year cumulative remnant gastric cancer incidence rates in the range of 1.4%‐6.8%.^6^, ^7^, ^8^ However, these previous reports were limited to patients with clinical or pathological T1 gastric cancer or those who survived for 5 years after surgery, even with advanced cancer. Compared to previous studies, the present study included more patients with different conditions, regardless of the stage of gastric cancer or survival status, which may be a relatively accurate reflection of clinical practice. The present study, which included approximately 30% of patients with advanced gastric cancer, showed similar 5‐year cumulative incidence rate of remnant gastric cancer.

Death is considered a competing risk in the analyses of cumulative incidence that included patients who died during the follow‐up period. In the Kaplan‐Meier and Cox regression analyses, patients with competitive risks should be excluded, as these methods overestimate the incidence of secondary cancer. In contrast, the competitive risk model enables the identification of incidence rates of and risk factors for secondary cancer in all patients, including those with advanced cancer.^18^, ^19^

In the present study, the 5‐year cumulative incidence rates of secondary laryngeal and esophageal cancers were 1.3%. Meanwhile, a previous study reported the 5‐year incidence rates of secondary upper gastrointestinal cancer after curative gastrectomy for gastric cancer to be approximately 0.15%,^20^ which suggests high incidence rates of secondary upper gastrointestinal cancer and high diagnostic performance of endoscopic examinations in the present study.

A Brinkman index ≥ 600, proximal gastrectomy, and tumor size ≥ 30 mm were identified as risk factors for remnant gastric cancer. Several reports have shown a high risk of remnant gastric cancer after proximal gastrectomy, with the 5‐year incidence rate of approximately 6.8%.^8^, ^21^ Another study reported that hypergastrinemia predisposed to gastric cancer^22^ and that hypergastrinemia induced by resection of the fundic gland region may increase the risk of remnant gastric cancer after proximal gastrectomy.^8^ Therefore, intensive follow‐up EGD may be recommended for patients after proximal gastrectomy. Smoking has been reported to increase the risk of gastric cancer and laryngeal cancer^23^; similarly, the present findings have shown that smoking increased the risk of remnant gastric cancer. No previous study has shown the tumor size of the initial gastric cancer to be a risk factor for remnant gastric cancer. A previous report showed that the depth of tumor invasion was a risk factor for remnant gastric cancer following gastrectomy for early gastric cancer but a tumor size ≥ 30 mm was not.^21^ Another report showed that a large size of the main lesion was a risk factor for missing multiple gastric cancers^24^; moreover, in the present study, some remnant gastric cancers might have been missed lesions during surgery. Patients with small gastric cancer might be less susceptible to tumor growth; hence, tumor size may be a confounding factor in this context. However, further consideration of the relationship between the tumor size of the gastric cancer and the development of remnant gastric cancer is necessary.

Patients with a history of gastric and esophageal cancer before gastrectomy were found to have a significantly higher incidence of secondary laryngeal and esophageal cancers. Patients with gastric cancer showed an increased risk for esophageal cancer,^13^ with an increased incidence of metachronous esophageal cancer after endoscopic resection for esophageal cancer (approximately 14%).^25^, ^26^ Therefore, patients with a history of upper gastrointestinal cancer before gastrectomy may be at high risk for secondary esophageal cancer.

All secondary upper gastrointestinal cancers detected during the follow‐up EGD were curatively resectable at diagnosis. Among patients with secondary cancer, 81.0% had endoscopically resectable cancer, with secondary cancer recurrence rates as low as 6.3%. Therefore, early detection of secondary upper gastrointestinal cancer during follow‐up EGD may improve overall survival.

Among 64 patients with secondary upper gastrointestinal cancer, a significantly higher proportion of habitual drinkers were observed in the regular follow‐up group than in the infrequent follow‐up group. One reason is that all the patients with a history of esophageal cancer, who were heavy drinkers, underwent annual follow‐up EGD. In addition, patients with detected secondary laryngeal and esophageal cancers, many of whom were heavy drinkers, also underwent annual follow‐up EGD after the diagnosis of the secondary laryngeal and esophageal cancers. Another speculation was that the attending surgeons might have recommended regular follow‐up EGD to patients that were heavy drinkers.

The proportion of endoscopically resectable cancer was significantly higher in the regular follow‐up group than in the infrequent follow‐up group, which supports the recommendations of the 2018 Japanese Gastric Cancer Treatment Guidelines. Treatment outcomes of endoscopic resection for early upper gastrointestinal cancer, even among patients with remnant gastric cancer, are overwhelmingly positive, although ESD for remnant gastric cancer is technically more demanding.^27^ Endoscopic treatment is associated with reduced abdominal symptoms and pain, and may promote good quality of life after treatment.^28^ Thus, early detection of secondary upper gastrointestinal cancer through frequent follow‐up EGD may help maintain patients' quality of life.

Several guidelines recommend follow‐up EGD after gastrectomy, but only a few define the optimum follow‐up duration and intervals between examinations.^29^, ^30^ The present findings suggest that regular follow‐up EGD may contribute to early detection of secondary upper gastrointestinal cancer and enable less invasive curative treatment; in addition, annual follow‐up EGD may benefit patients with risk factors for secondary upper gastrointestinal cancer.

The current study had several limitations. First, we did not analyze the cost‐effectiveness of follow‐up EGD. Given that EGD is relatively inexpensive in Japan, the cost may not restrict clinical implementation. Secondly, we could not collect information regarding *Helicobacter pylori* infection, which is a known risk factor for gastric cancer.^31^ Our hospital does not routinely assess for *Helicobacter pylori* infection before gastrectomy; therefore, atrophic gastritis before initial surgery was substituted for *Helicobacter pylori* infection.

## 5 CONCLUSIONS

The current study revealed 5‐year cumulative incidence rates of 2.9% for remnant gastric cancer and 1.3% for secondary laryngeal and esophageal cancers. Risk factors for remnant gastric cancer included heavy smoking, proximal gastrectomy, and gastric tumor size ≥ 30 mm; risk factors for secondary laryngeal and esophageal cancers included a history of upper gastrointestinal cancer before gastrectomy. Curative treatment was performed for almost all cancers, indicating the effectiveness of follow‐up EGD after gastrectomy for the early detection of secondary upper gastrointestinal cancer. Annual follow‐up EGD may benefit patients presenting with identified risk factors for secondary upper gastrointestinal cancer.

## DISCLOSURE

Conflict of Interest: Authors declare no conflict of interests for this article. None of the authors are editors or editorial board member of AGS.

Ethical Approval: The Institutional Review Board of Shizuoka Cancer Center approved the present study (approval no. J2020‐94‐2020‐1), which conformed with the Declaration of Helsinki. Patient consent for participation was obtained using the opt‐out method.

## Supporting information

Fig S1

Fig S2

Table S1‐S4
