# Supplementary figures and images for: Detection of secondary upper gastrointestinal tract cancer during follow‐up esophagogastroduodenoscopy after gastrectomy for gastric cancer
Source: Ann Gastroenterol Surg. 2022 Jan 25;6(4):486–95. doi: 10.1002/ags3.12546 (PMC9271028; doi:10.1002/ags3.12546)

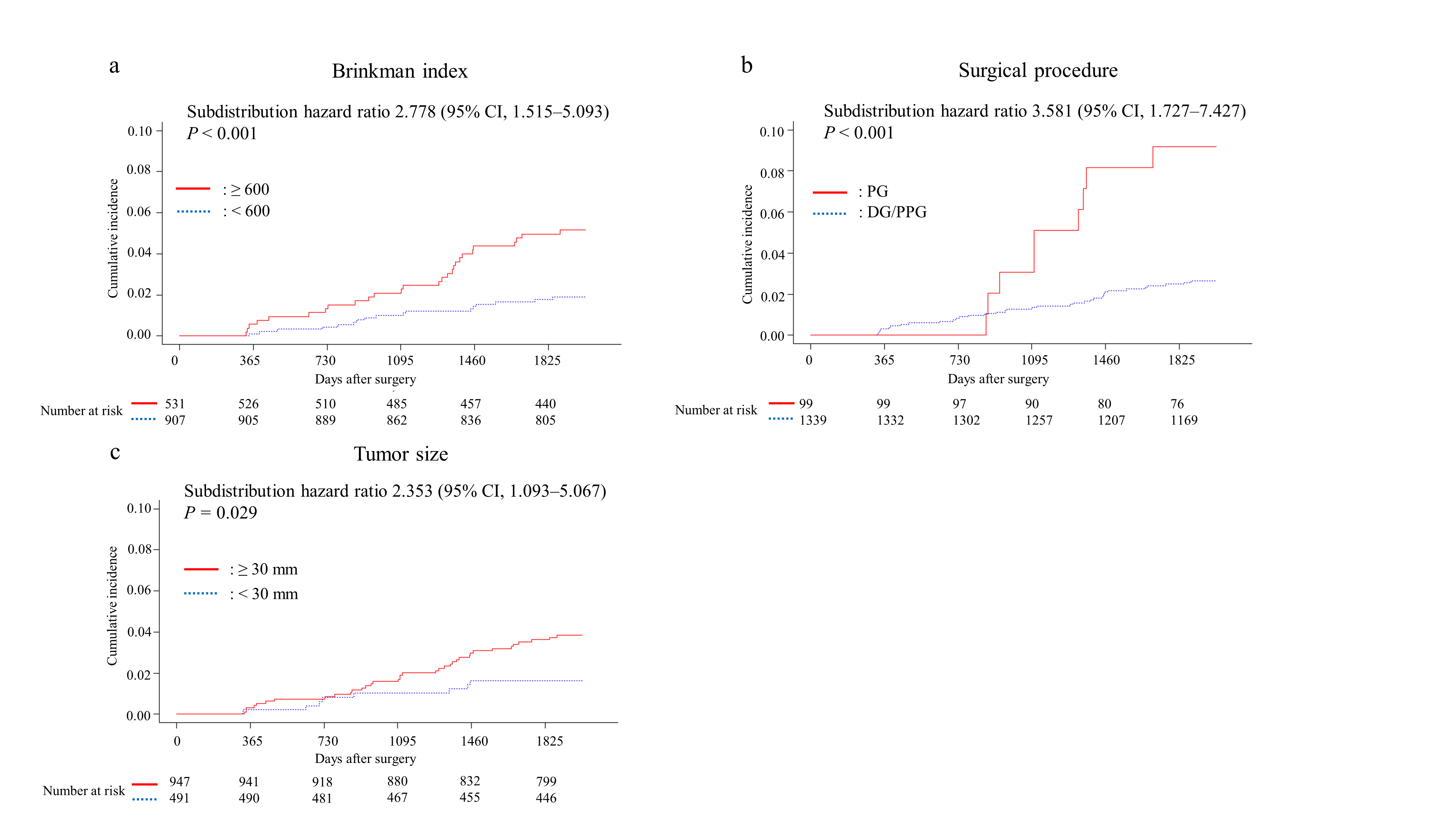

Supplement: Supplementary file 1 — Fig S1 [file AGS3-6-486-s002.tif]

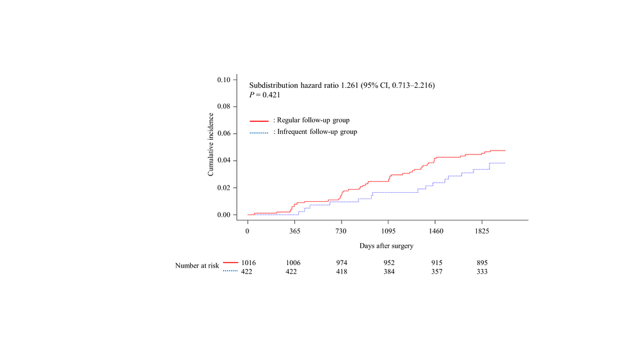

Supplement: Supplementary file 2 — Fig S2 [file AGS3-6-486-s003.docx]
