# Supplementary material for: Detection of secondary upper gastrointestinal tract cancer during follow‐up esophagogastroduodenoscopy after gastrectomy for gastric cancer
Source: Ann Gastroenterol Surg. 2022 Jan 25;6(4):486–95. doi: 10.1002/ags3.12546 (PMC9271028; doi:10.1002/ags3.12546)
Supplement: Supplementary file 3 — Table S1‐S4 [file AGS3-6-486-s001.docx]

**Supplemental Table 1**: Risk factors for secondary laryngeal and esophageal cancers

|  |  |  | Univariate analysis |  |  |  | Multivariate analysis |  |  |
| --- | --- | --- | --- | --- | --- | --- | --- | --- | --- |
|  | Number of patients |  | Subdistribution hazard ratio (95% CI ^†^) |  |  |  | Subdistribution hazard ratio (95% CI ^†^) |  |  |
|  |  |  |  |  | *P* |  |  |  | *P* |
| Sex |  |  |  |  |  |  |  |  |  |
| Female | 475 |  | 1 |  |  |  | 1 |  |  |
| Male | 963 |  | 9.984 (1.339–74.416) |  | 0.025 |  | 4.198 (0.480–36.748) |  | 0.195 |
| Age (years) |  |  |  |  |  |  |  |  |  |
| < 70 | 877 |  | 1 |  |  |  |  |  |  |
| ≥ 70 | 561 |  | 0.490 (0.179–1.338) |  | 0.164 |  |  |  |  |
| Brinkman index |  |  |  |  |  |  |  |  |  |
| < 600 | 907 |  | 1 |  |  |  | 1 |  |  |
| ≥ 600 | 531 |  | 2.312 (0.975–5.482) |  | 0.057 |  | 1.277 (0.464–3.514) |  | 0.636 |
| Alcohol |  |  |  |  |  |  |  |  |  |
| Nondrinker or social drinker | 725 |  | 1 |  |  |  | 1 |  |  |
| Habitual drinker | 713 |  | 6.182 (1.822–20.980) |  | 0.003 |  | 3.168 (0.981–10.231) |  | 0.054 |
| History of gastric cancer before gastrectomy |  |  |  |  |  |  |  |  |  |
| No | 1413 |  | 1 |  |  |  | 1 |  |  |
| Yes | 25 |  | 9.967 (2.927–33.938) |  | < 0.001 |  | 4.937 (1.630–14.950) |  | 0.005 |
| History of esophageal cancer |  |  |  |  |  |  |  |  |  |
| No | 1419 |  | 1 |  |  |  | 1 |  |  |
| Yes | 19 |  | 37.342 (14.241–97.916) |  | < 0.001 |  | 17.736 (6.533–48.153) |  | < 0.001 |
| Atrophic gastritis before initial surgery |  |  |  |  |  |  |  |  |  |
| Mild or no atrophy (C-0, C-1, C-2) | 264 |  | 1 |  |  |  |  |  |  |
| Moderate or severe (C-3, O-1, O-2, O-3) | 1174 |  | 4.506 (0.607–33.433) |  | 0.141 |  |  |  |  |
| Synchronous multiple gastric cancer |  |  |  |  |  |  |  |  |  |
| No | 1253 |  | 1 |  |  |  |  |  |  |
| Yes | 185 |  | 1.592 (0.538–4.711) |  | 0.401 |  |  |  |  |
| Surgical procedure |  |  |  |  |  |  |  |  |  |
| Distal gastrectomy/Pylorus preserving gastrectomy | 1339 |  | 1 |  |  |  |  |  |  |
| Proximal gastrectomy | 99 |  | 0.678 (0.091–5.071) |  | 0.705 |  |  |  |  |
| Pathohistology of main initial cancer |  |  |  |  |  |  |  |  |  |
| Differentiated type | 753 |  | 1 |  |  |  |  |  |  |
| Undifferentiated/Special type | 685 |  | 1.003 (0.426–2.360) |  | 0.995 |  |  |  |  |
| Tumor size |  |  |  |  |  |  |  |  |  |
| < 30 mm | 491 |  | 1 |  |  |  | 1 |  |  |
| ≥ 30 mm | 947 |  | 0.385 (0.162–0.914) |  | 0.030 |  | 0.536 (0.221–1.301) |  | 0.168 |
| Depth of tumor invasion |  |  |  |  |  |  |  |  |  |
| T0/T1 | 1004 |  | 1 |  |  |  |  |  |  |
| T2/T3/T4 | 434 |  | 0.924 (0.358–2.380) |  | 0.869 |  |  |  |  |
| Lymphatic metastasis |  |  |  |  |  |  |  |  |  |
| Negative | 1008 |  | 1 |  |  |  |  |  |  |
| Positive | 430 |  | 0.733 (0.269–2.001) |  | 0.544 |  |  |  |  |
| Pathological stage |  |  |  |  |  |  |  |  |  |
| 0–I | 1042 |  | 1 |  |  |  |  |  |  |
| II–IV | 396 |  | 1.321 (0.533–3.272) |  | 0.547 |  |  |  |  |
| Adjuvant chemotherapy |  |  |  |  |  |  |  |  |  |
| No | 1171 |  | 1 |  |  |  |  |  |  |
| Yes | 267 |  | 1.029 (0.346–3.056) |  | 0.959 |  |  |  |  |

^†^ CI confidence interval

**Supplemental Table 2**: Clinicopathological characteristics of the patients in the regular and infrequent follow-up groups

|  | Total |  | Regular follow-up |  | Infrequent follow-up |  |  |
| --- | --- | --- | --- | --- | --- | --- | --- |
|  |  |  |  |  |  |  |  |
|  | n = 1438 |  | n = 1016 |  | n = 422 |  | *P* |
| Sex |  |  |  |  |  |  |  |
| Male | 963 (67.0%) |  | 667 (65.6%) |  | 296 (70.1%) |  |  |
| Female | 475 (33.0%) |  | 349 (34.4%) |  | 126 (29.9%) |  | 0.109 |
| Age (years) |  |  |  |  |  |  |  |
| ≥ 70 | 561 (39.0%) |  | 357 (35.1%) |  | 204 (48.3%) |  |  |
| < 70 | 877 (61.0%) |  | 659 (64.9%) |  | 218 (51.7%) |  | < 0.001 |
| Brinkman index |  |  |  |  |  |  |  |
| ≥ 600 | 531 (36.9%) |  | 363 (35.7%) |  | 168 (39.8%) |  |  |
| < 600 | 907 (63.1%) |  | 653 (64.3%) |  | 254 (60.2%) |  | 0.150 |
| Alcohol |  |  |  |  |  |  |  |
| Habitual drinker | 713 (49.6%) |  | 520 (51.2%) |  | 193 (45.7%) |  |  |
| Nondrinker or social drinker | 725 (50.4%) |  | 496 (48.8%) |  | 229 (54.3%) |  | 0.064 |
| History of gastric cancer before gastrectomy |  |  |  |  |  |  |  |
| Yes | 25 (1.7%) |  | 17 (1.7%) |  | 8 (1.9%) |  |  |
| No | 1413 (98.3%) |  | 999 (98.3%) |  | 414 (98.1%) |  | 0.825 |
| History of esophageal cancer |  |  |  |  |  |  |  |
| Yes | 19 (1.3%) |  | 16 (1.6%) |  | 3 (0.7%) |  |  |
| No | 1419 (98.7%) |  | 1000 (98.4%) |  | 419 (99.3%) |  | 0.309 |
| Atrophic gastritis before initial surgery |  |  |  |  |  |  |  |
| Moderate or severe (C-3, O-1, O-2, O-3) | 1174 (81.6%) |  | 821 (80.8%) |  | 353 (83.6%) |  |  |
| Mild or no atrophy (C-0, C-1, C-2) | 264 (18.4%) |  | 195 (19.2%) |  | 69 (16.4%) |  | 0.231 |
| Synchronous multiple gastric cancer |  |  |  |  |  |  |  |
| Yes | 185 (12.9%) |  | 136 (13.4%) |  | 49 (11.6%) |  |  |
| No | 1253 (87.1%) |  | 880 (86.6%) |  | 373 (88.4%) |  | 0.388 |
| Surgical procedure |  |  |  |  |  |  |  |
| Distal gastrectomy/Pylorus preserving gastrectomy | 1339 (93.1%) |  | 942 (92.7%) |  | 397 (94.1%) |  |  |
| Proximal gastrectomy | 99 (6.9%) |  | 74 (7.3%) |  | 25 (5.9%) |  | 0.423 |
| Pathohistology of main initial cancer |  |  |  |  |  |  |  |
| Differentiated type | 753 (52.4%) |  | 512 (50.4%) |  | 241 (57.1%) |  |  |
| Undifferentiated/Special type | 685 (47.6%) |  | 504 (49.6%) |  | 181 (42.9%) |  | 0.021 |
| Tumor size |  |  |  |  |  |  |  |
| ≥ 30 mm | 947 (65.9%) |  | 663 (65.3%) |  | 284 (67.3%) |  |  |
| < 30 mm | 491 (34.1%) |  | 353 (34.7%) |  | 138 (32.7%) |  | 0.464 |
| Depth of tumor invasion |  |  |  |  |  |  |  |
| T0/T1 | 1004 (69.8%) |  | 727 (71.6%) |  | 277 (65.6%) |  |  |
| T2/T3/T4 | 434 (30.2%) |  | 289 (28.4%) |  | 145 (34.4%) |  | 0.027 |
| Lymphatic metastasis |  |  |  |  |  |  |  |
| Positive | 430 (29.9%) |  | 284 (28.0%) |  | 146 (34.6%) |  |  |
| Negative | 1008 (70.1%) |  | 732 (72.0%) |  | 276 (65.4%) |  | 0.014 |
| Pathological stage |  |  |  |  |  |  |  |
| 0–I | 1042 (72.5%) |  | 749 (73.7%) |  | 293 (69.4%) |  |  |
| II–IV | 396 (27.5%) |  | 267 (26.3%) |  | 129 (30.6%) |  | 0.105 |
| Adjuvant chemotherapy |  |  |  |  |  |  |  |
| Yes | 267 (18.6%) |  | 187 (18.4%) |  | 80 (19.0%) |  |  |
| No | 1171 (81.4%) |  | 829 (81.6%) |  | 342 (81.0%) |  | 0.823 |

**Supplemental Table 3**: Clinicopathological characteristics of the patients with secondary upper gastrointestinal cancer in the regular and infrequent follow-up groups

|  | Total |  | Regular follow-up |  | Infrequent follow-up |  |  |
| --- | --- | --- | --- | --- | --- | --- | --- |
|  |  |  |  |  |  |  |  |
|  | n = 64 |  | n = 48 |  | n = 16 |  | *P* |
| Sex |  |  |  |  |  |  |  |
| Male | 56 (87.5%) |  | 43 (89.6%) |  | 13 (81.3%) |  |  |
| Female | 8 (12.5%) |  | 5 (10.4%) |  | 3 (18.7%) |  | 0.401 |
| Age (years) |  |  |  |  |  |  |  |
| ≥ 70 | 26 (40.6%) |  | 18 (37.5%) |  | 8 (50.0%) |  |  |
| < 70 | 38 (59.4%) |  | 30 (62.5%) |  | 8 (50.0%) |  | 0.396 |
| Brinkman index |  |  |  |  |  |  |  |
| ≥ 600 | 39 (60.9%) |  | 31 (64.6%) |  | 8 (50.0%) |  |  |
| < 600 | 25 (39.1%) |  | 17 (35.4%) |  | 8 (50.0%) |  | 0.379 |
| Alcohol |  |  |  |  |  |  |  |
| Habitual drinker | 37 (57.8%) |  | 32 (66.7%) |  | 5 (31.2%) |  |  |
| Nondrinker or social drinker | 27 (42.2%) |  | 16 (33.3%) |  | 11 (68.8%) |  | 0.019 |
| History of gastric cancer before gastrectomy |  |  |  |  |  |  |  |
| Yes | 4 (6.2%) |  | 3 (6.2%) |  | 1 (6.2%) |  |  |
| No | 60 (93.8%) |  | 45 (93.8%) |  | 15 (93.8%) |  | 1 |
| History of esophageal cancer |  |  |  |  |  |  |  |
| Yes | 6 (9.4%) |  | 6 (12.5%) |  | 0 (0%) |  |  |
| No | 58 (90.6%) |  | 42 (87.5%) |  | 16 (100.0%) |  | 0.323 |
| Atrophic gastritis before initial surgery |  |  |  |  |  |  |  |
| Moderate or severe (C-3, O-1, O-2, O-3) | 61 (95.3%) |  | 47 (97.9%) |  | 14 (87.5%) |  |  |
| Mild or no atrophy (C-0, C-1, C-2) | 3 (4.7%) |  | 1 (2.1%) |  | 2 (12.5%) |  | 0.152 |
| Synchronous multiple gastric cancer |  |  |  |  |  |  |  |
| Yes | 12 (18.7%) |  | 9 (18.7%) |  | 3 (18.7%) |  |  |
| No | 52 (81.3%) |  | 39 (81.3%) |  | 13 (81.3%) |  | 1 |
| Surgical procedure |  |  |  |  |  |  |  |
| Distal gastrectomy/Pylorus preserving gastrectomy | 54 (84.4%) |  | 39 (81.3%) |  | 15 (93.8%) |  |  |
| Proximal gastrectomy | 10 (15.6%) |  | 9 (18.7%) |  | 1 (6.2%) |  | 0.429 |
| Pathohistology of main initial cancer |  |  |  |  |  |  |  |
| Differentiated type | 41 (64.1%) |  | 30 (62.5%) |  | 11 (68.8%) |  |  |
| Undifferentiated/Special type | 23 (35.9%) |  | 18 (37.5%) |  | 5 (31.2%) |  | 0.768 |
| Tumor size |  |  |  |  |  |  |  |
| ≥ 30 mm | 44 (68.8%) |  | 30 (62.5%) |  | 14 (87.5%) |  |  |
| < 30 mm | 20 (31.2%) |  | 18 (37.5%) |  | 2 (12.5%) |  | 0.071 |
| Depth of tumor invasion |  |  |  |  |  |  |  |
| T0/T1 | 40 (62.5%) |  | 31 (64.6%) |  | 9 (56.3%) |  |  |
| T2/T3/T4 | 24 (37.5%) |  | 17 (35.4%) |  | 7 (43.7%) |  | 0.565 |
| Lymphatic metastasis |  |  |  |  |  |  |  |
| Positive | 17 (26.6%) |  | 10 (20.8%) |  | 7 (43.7%) |  |  |
| Negative | 47 (73.4%) |  | 38 (79.2%) |  | 9 (56.3%) |  | 0.103 |
| Pathological stage |  |  |  |  |  |  |  |
| 0–I | 44 (68.8%) |  | 35 (72.9%) |  | 9 (56.3%) |  |  |
| II–IV | 20 (31.2%) |  | 13 (27.1%) |  | 7 (43.7%) |  | 0.229 |
| Adjuvant chemotherapy |  |  |  |  |  |  |  |
| Yes | 9 (14.1%) |  | 6 (12.5%) |  | 3 (18.7%) |  |  |
| No | 55 (85.9%) |  | 42 (87.5%) |  | 13 (81.3%) |  | 0.679 |

**Supplemental Table 4**: Details of secondary upper gastrointestinal cancer and endoscopically resectability in the regular and infrequent follow-up groups

|  | Total |  | Regular follow-up |  | Infrequent follow-up |  |  |
| --- | --- | --- | --- | --- | --- | --- | --- |
|  | n = 63 |  | n = 48 |  | n = 15 |  | *P* |
| Patients with endoscopically resectable cancer | 51 (81.0%) |  | 42 (87.5%) |  | 9 (60.0%) |  |  |
| Patients with endoscopically unresectable cancer | 12 (19.0%) |  | 6 (12.5%) |  | 6 (40%) |  | 0.028 |
|  |  |  |  |  |  |  |  |
| Details of secondary upper gastrointestinal cancer^†^  (Endoscopically resectable/unresectable) |  |  |  |  |  |  |  |
| Remnant gastric cancer | 43 (33/10) |  | 30 (25/5) |  | 13 (8/5) |  |  |
| pT1a | 33 (32/1) |  | 24 (24/0) |  | 9 (8/1) |  |  |
| pT1b | 6 (1/5) |  | 4 (1/3) |  | 2 (0/2) |  |  |
| pT2-4 | 4 (0/4) |  | 2 (0/2) |  | 2 (0/2) |  |  |
| Laryngeal cancer | 8 (8/0) |  | 6 (6/0) |  | 2 (2/0) |  |  |
| Esophageal cancer | 15 (13/2) |  | 13 (12/1) |  | 2 (1/1) |  |  |
| T1a | 13 (13/0) |  | 12 (12/0) |  | 1 (1/0) |  |  |
| T1b | 2 (0/2) |  | 1 (0/1) |  | 1 (0/1) |  |  |

^†^ Duplicated
